# Low-Temperature Dyeing of Polylactic Acid Fabrics with Microbial Prodigiosin Enabled by Natural Deep Eutectic Solvent Treatment

**DOI:** 10.3390/polym18101160

**Published:** 2026-05-08

**Authors:** Lili Feng, Shaoxin Dong, Xuetong Wang, Yu Han, Hongjie Zhang

**Affiliations:** 1 College of Textile and Apparel, Quanzhou Normal University, Quanzhou 362000, China; fll827@qztc.edu.cn (L.F.);; 2 Key Laboratory of Clothing Materials of Universities in Fujian, Quanzhou Normal University, Quanzhou 362000, China

**Keywords:** polylactic acid, natural deep eutectic solvent, low-temperature dyeing, microbial prodigiosin, sustainable textiles

## Abstract

Polylactic acid (PLA), a biodegradable polymer derived from renewable resources, represents a promising candidate for sustainable textiles. Nevertheless, its practical application remains limited by the requirement for high-temperature dyeing, which can induce polymer hydrolysis and lead to the loss of fiber strength. To address this limitation, PLA fabric was treated with an eco-friendly natural deep eutectic solvent (NaDES) composed of glycerol and citric acid. The treatment was found to enhance fiber surface roughness and internal looseness, which facilitated dye diffusion and allowed for a significant reduction in dyeing temperature. When dyed with microbial prodigiosin, the treated PLA fabric achieved a color depth at 70 °C that was equivalent to untreated fabric at 90 °C, while also exhibiting a 93.56% bacteriostatic rate against *Staphylococcus aureus* due to the inherent antibacterial property of microbial prodigiosin. This work provides a novel and sustainable strategy for the eco-friendly dyeing of PLA textiles.

## 1. Introduction

Polylactic acid (PLA) fiber is a bio-based synthetic polymer derived from renewable resources including corn and cassava [1,2,3]. Owing to its biodegradability and environmental friendliness, PLA is widely recognized as a sustainable material. Moreover, its favorable mechanical strength, optical clarity and processability make PLA a prominent alternative to petroleum-based synthetic fibers [4,5,6], with growing applications in textiles, medical materials, and packaging [7,8,9,10]. Driven by growing demand, global PLA production capacity is expanding rapidly, supported by major manufacturing bases established in North America, Europe, and Asia.

However, the hydrophobic nature of polylactic acid (PLA) results in poor dye affinity, which represents a significant barrier to its textile application [11]. High-temperature dyeing processes are required to achieve sufficient dye uptake but inevitably cause ester bond hydrolysis and polymer degradation [12,13,14,15]. To address this issue, low-temperature dyeing strategies have emerged to reduce the dyeing temperature and the hydrolytic degradation of PLA fibers, which are primarily realized through two approaches. One approach focuses on fiber modification, which involves physical treatments such as plasma treatment, ultraviolet irradiation [16,17], as well as chemical modifications including graft polymerization, copolymerization, and nanoparticle incorporation during melt spinning [18,19]. These methods aim to roughen the fiber surface, introduce polar groups, or reduce crystallinity, thereby lowering dye diffusion resistance and improving dyeing performance under low-temperature conditions. Although these methods have been widely investigated, they remain limited by their high cost and process complexity. The second approach involves the development of novel low-temperature dyeing systems, such as using an environmentally friendly solvent to swell the fibers or employing non-aqueous dyeing media to avoid the hydrolysis of PLA fibers under high-temperature conditions [20,21].

Deep eutectic solvents (DES) represent a class of eutectic mixtures formed via hydrogen bonding interactions between a hydrogen bond donor (HBD) and a hydrogen bond acceptor (HBA) [22,23,24]. They are characterized by a low melting point and tunable physicochemical properties. Typical HBDs include compounds like urea, organic acids, and polyols, whereas common HBAs include quaternary ammonium salts such as choline chloride. DES exhibit many advantageous characteristics similar to ionic liquids, yet they generally feature simpler preparation processes and are more cost effective. As a result, DES have been widely utilized in various fields, including chemical synthesis, product extraction and textile processing [25,26,27,28,29]. In recent years, the application of DES has been successfully extended to fiber modification [30,31,32,33,34]. For instance, a thymol/coumarin-based DES enabled PLA fabrics to achieve comparable color depth at 70 °C to untreated fabrics at 90 °C, while a choline chloride/oxalic acid DES enhanced the surface roughness and chemical activity of PLA filaments, allowing them to achieve comparable dyeing performance at 90 °C to untreated filaments at 110 °C [31,34].

**Figure 1:**
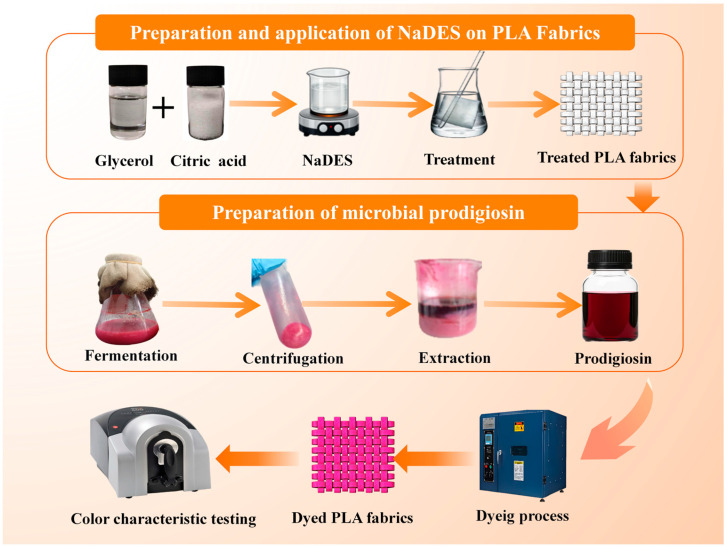
The experimental process for preparation of NaDES, treatment of PLA fabrics and dyeing of PLA fabrics with microbial prodigiosin.

This study aimed to develop a green treatment strategy to enable the low-temperature dyeing of PLA fabrics, specifically for applying natural dyes that often exhibit poor thermal stability [35,36], such as microbial prodigiosin. Natural deep eutectic solvents (NaDES), a class of deep eutectic solvents derived from natural components, are generally considered to be eco-friendly. Accordingly, a uniform NaDES based on glycerol and citric acid was prepared and applied to treat PLA fabrics, after which the effect of this treatment on the dyeing efficiency of microbial prodigiosin was systematically investigated, as illustrated in Figure 1. Furthermore, the underlying modification mechanism of the NaDES on PLA fibers was elucidated through comprehensive characterizations and measurements of the treated PLA fabrics. This strategy offers a technological pathway toward the green production of PLA textiles.

## 2. Materials and Methods

### 2.1. Materials

Yeast extract and tryptone were purchased from Sangon Biotech Co., Ltd. (Shanghai, China). Chemical reagents such as glycerol and citric acid were procured from Aladdin Bio-Chem Technology Co., Ltd. (Shanghai, China). Peanut powder was obtained from a local agricultural source. The bacterial strains *Serratia marcescens* was obtained from the China Center of Industrial Culture Collection, while the bacterial strains *Escherichia coli* and *Staphylococcus aureus* were obtained from the American Type Culture Collection. The polylactic acid (PLA) fabrics used in this study (weight 140 g/m^2^, warp 62 yarns per inch, weft 268 yarns per inch) were supplied by Zibo Pueridi Textile Technology Co., Ltd. (Zibo, China).

### 2.2. Methods

#### 2.2.1. NaDES Preparation and PLA Fabric Treatment

The NaDES was prepared by combining glycerol and citric acid at a specific molar ratio, followed by heating at 90 °C for 3 h with continuous stirring until a homogeneous and transparent liquid formed. The PLA fabrics were then immersed in the NaDES at varying bath ratios. After treatment at a specific temperature and duration, the fabrics were removed, subsequently washed with deionized water, and finally dried. For subsequent experiments, a series of representative samples were prepared, which included the original PLA fabric (OP), and NaDES-treated samples prepared at 50, 60, 70, 80, and 90 °C (TP1–TP5).

#### 2.2.2. Preparation of Microbial Prodigiosin

For pre-cultivation, a single clone of *S. marcescens* was inoculated into 50 mL of LB medium containing 10 g/L tryptone, 5 g/L yeast extract and 10 g/L sodium chloride, which was incubated at 28 °C with shaking at 160 rpm for approximately 12 h. This seed culture was then transferred into a 250 mL shake flask containing 100 mL of fermentation medium with 2% (*w*/*v*) peanut powder. Following 72 h of fermentation under the same conditions (28 °C, 160 rpm), the culture broth was centrifuged at 10,000 rpm for 10 min to harvest the cells. The resulting pellet was extracted with ethyl acetate, and the pigment-rich organic layer was collected as the prodigiosin extract for subsequent dyeing applications.

#### 2.2.3. Low-Temperature Dyeing

The dyeing performance of both the original and NaDES-treated PLA fabrics was evaluated using microbial prodigiosin as the bio-colorant. The dyeing process was carried out under the following conditions: prodigiosin concentration of 6.0% (*v*/*v*), bath ratio of 1:50 (fabric mass to dye liquor volume), dyeing duration of 30 min, and dyeing temperatures ranging from 60 to 90 °C. After dyeing, the PLA fabrics were thoroughly rinsed with deionized water and dried for subsequent characterization.

### 2.3. Characterization

Scanning electron microscopy (SEM): The surface morphology of PLA samples was characterized using a scanning electron microscope (TESCAN MIRA LMS, Brno, Czech Republic). To enhance surface conductivity for high-quality imaging, all samples were sputter-coated with a thin gold layer prior to examination. Micrographs were systematically captured at a standardized magnification of 3000× to ensure comparative evaluation of surface features across different samples.

Fourier Transform Infrared (FTIR) spectra: The infrared spectra of the samples were measured on a Nicolet iS10 Infrared Raman spectrometer (Thermo Fisher, Waltham, MA, USA) within a wavelength range of 4000–800 cm^−1^.

X-ray photoelectron spectra (XPS): The XPS of the fabrics were obtained on a PHI 5000 VersaProbe III X-ray photoelectron spectrometer (ULVAC-PHI, Chigasaki, Japan), employing a monochromatized Al Kα source operating at 15 kV and 50 W.

The X-ray diffraction spectra (XRD): XRD patterns of the samples were obtained using a Bruker D8 Advance diffractometer (Bruker AXS, Karlsruhe, Germany) employing Cu Kα radiation at 40 kV and 40 mA, with scattering angles ranging from 2° to 40°.

Color characteristics: The color characteristics of the dyed PLA fabrics were evaluated using a Datacolor 800 spectrophotometer (Datacolor company, Lawrenceville, GA, USA) under a D65 standard illuminant with a 10° standard observer. Measurements were taken at five random locations on each fabric sample, and the reported values represent the mean of these replicate measurements.

Antibacterial property: The antibacterial properties of the samples against *S. aureus* and *E. coli* was evaluated according to the textile antibacterial standard GB/T 20944.3-2008.

## 3. Results

### 3.1. Characterization of the NaDES

**Figure 2:**
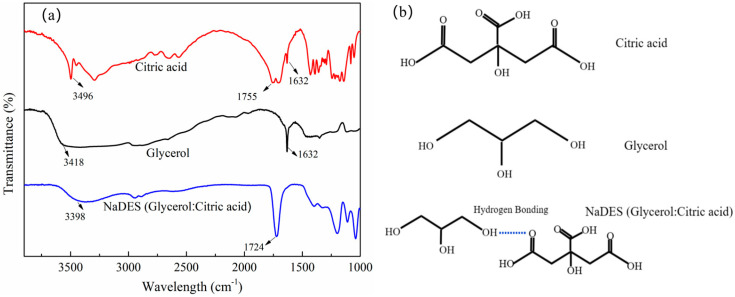
The FTIR of citric acid, glycerol and NaDES (**a**); chemical structure and hydrogen bonding of citric acid and glycerol (**b**).

FTIR analysis was conducted on the freshly prepared NaDES to investigate potential chemical bond changes by comparing its spectrum with those of pure citric acid and glycerol. A glycerol-to-citric acid molar ratio of 2:1 was selected to promote a continuous liquid phase and avoid solid crystallization. As shown in Figure 2a, the FTIR spectrum of citric acid exhibited prominent peaks at 1755 cm^−1^, corresponding to the stretching vibrations of its carbonyl (C=O) groups [37]. In the glycerol spectrum, distinct peaks were observed at 3418 cm^−1^ and 1632 cm^−1^, corresponding to O–H stretching and bending vibrations, respectively. The FTIR analysis revealed that the characteristic peaks of NaDES mainly originated from the overlapping of glycerol and citric acid. The O–H bending vibration of glycerol shifted from 3418 cm^−1^ to 3398 cm^−1^, whereas the C=O stretching vibration of citric acid shifted from 1755 cm^−1^ to 1724 cm^−1^. Considering that in the NaDES system composed of glycerol and citric acid, the -OH group in glycerol serves as a hydrogen donor, while the C=O group in citric acid acts as a hydrogen acceptor, the shifts in the C=O and -OH bands of the NaDES are regarded as the formation of hydrogen bond, as depicted in Figure 2b.

### 3.2. Characterization of the NaDES-Treated PLA Fabric

#### 3.2.1. Morphology

**Figure 3:**
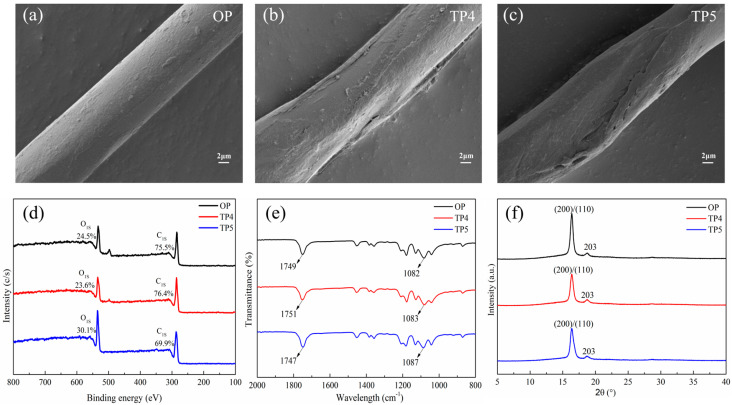
Characterization of NaDES-treated PLA fabric. SEM graph of OP (**a**), TP4 (**b**) and TP5 (**c**); the XPS (**d**) FTIR (**e**) and XRD (**f**) results of PLA samples.

The alterations in the surface morphology of the original and NaDES treated PLA fabrics are presented in Figure 3a–c. It is obvious that the original PLA fibers typically exhibit a smooth and uniform surface, which limits the adsorption and diffusion of dye molecules, resulting in the inferior dyeability of PLA, particularly under low-temperature conditions. Nevertheless, subsequent to the treatment with NaDES, the surface of PLA fiber became rough with distinct grooves and etching. This morphological change can be ascribed to synergistic interactions between NaDES components and PLA polymer chains. Glycerol and citric acid disrupt the ordered arrangement of surface molecular chains through hydrogen bonding, inducing localized swelling and physical perturbation that lead to the formation of wrinkles. Meanwhile, the acidity of citric acid catalyzes the partial hydrolysis of surface ester bonds, resulting in a certain degree of etching on the fiber surface. These synergistic effects collectively increase the surface roughness of PLA fibers, which in turn facilitates dye adsorption.

#### 3.2.2. XPS Analysis

XPS analysis in Figure 3d indicates that the contents of carbon (C) and oxygen (O) in the original PLA fabric were 75.5% and 24.5%, respectively. After treatment with the NaDES at 80 °C, the elemental composition exhibited minimal change, suggesting a relatively mild impact under this condition. However, treatment with the NaDES at 90 °C markedly altered the surface elemental composition of PLA fabric, as evidenced by a decrease in C content from 75.5% to 69.9% and a corresponding increase in O content from 24.5% to 30.1%. Consequently, the O/C atomic ratio rose significantly from 0.32 to 0.43. These alterations evidently demonstrate a more robust chemical interaction between the NaDES and the PLA surface at elevated temperatures, during which the cleavage of ester bonds in the molecular chains generated oxygen-containing functional groups including carboxyl (-COOH) and hydroxyl (-OH) groups, thereby increasing the relative oxygen content on the surface of the PLA fabrics.

#### 3.2.3. FTIR of Fabrics Analysis

The FTIR spectra of the original and NaDES-treated PLA fabrics are presented in Figure 3e. The spectrum of the original PLA exhibits its characteristic absorption peaks. Specifically, the peaks at 1749 cm^−1^ and 1082 cm^−1^ correspond to the C=O and C–O–C stretching vibrations of the ester group, respectively [38,39]. The FTIR spectrum of NaDES-treated PLA exhibited no new characteristic absorption peaks, indicating that NaDES treatment did not alter the fundamental chemical structure of PLA or introduce new functional groups within the bulk polymer. However, at a treatment temperature of 80 °C, NaDES adsorbed onto the surface of the PLA fibers, inducing physical perturbation of the exposed C=O groups and resulting in a slight shift from 1749 cm^−1^ to 1751 cm^−1^. In contrast, the C–O–C bonds within the polymer backbone remained largely unaffected, as reflected by the negligible change in its characteristic peak position. When the treatment temperature was raised to 90 °C, the NaDES fully penetrated into the amorphous regions of the PLA fibers. Hydrogen bonds formed between the hydroxyl groups of the NaDES and the ester carbonyl groups of PLA, shifting the C=O absorption band to 1747 cm^−1^. Meanwhile, the penetration of the NaDES induced swelling of the PLA molecular chains and disrupted the ordered arrangement of the polymer backbone, which in turn caused a shift of the C–O–C absorption band to 1087 cm^−1^.

#### 3.2.4. XRD Analysis

The XRD patterns of OP, TP4, and TP5 are presented in Figure 3f. The characteristic diffraction peaks of the α-crystal form of PLA are typically located at approximately 16.4° and 18.6°, corresponding to the (200)/(110) and (203) crystal planes, respectively [40]. Notably, the XRD profiles of TP4 and TP5 remain largely similar to that of OP, and no additional diffraction peaks were observed after NaDES treatment, indicating that the treatment did not induce the formation of new crystalline phases. Nevertheless, a reduction in the intensity and integrated area of the diffraction peaks corresponding to the (200)/(110) and (203) planes were observed after the treatment. This indicates a decrease in the crystallinity and a corresponding increase in the amorphous phase content within the PLA fibers. It is proposed that the NaDES components penetrate the interior of the PLA fibers and disrupt the ordered arrangement of polymer chains through hydrogen bonding. This structural alteration expands the proportion and accessibility of amorphous domains, thereby promoting the diffusion of dye molecules into the interior of the fibers.

### 3.3. Optimization of NaDES Treatment for PLA Dyeing

**Figure 4:**
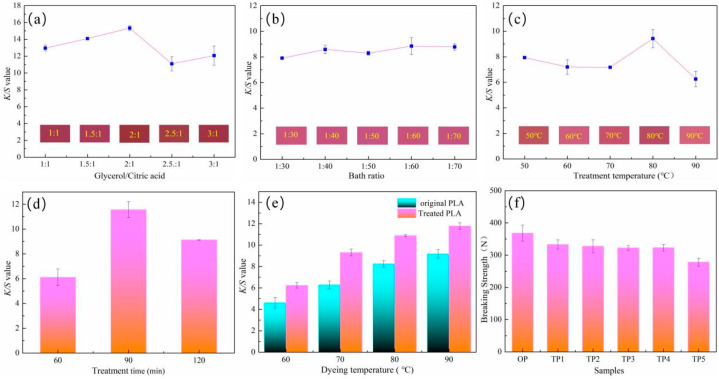
The influence of glycerol/citric acid molar ratio (**a**), bath ratio (**b**), treatment temperature (**c**), treatment time (**d**), and dyeing temperature (**e**) on *K/S* value, and the effect of treatment temperature on breaking strength (**f**).

In this study, a systematic investigation was performed to study the influence of NaDES treatment conditions on the dyeing performance of PLA fabrics, which was quantified by the *K/S* value. The optimization of key parameters, including the molar ratio of NaDES components, bath ratio, treatment temperature, and treatment time, is presented in Figure 4.

Figure 4a,b illustrate the effect of the NaDES components’ molar ratio and bath ratio on the *K/S* value. A non-linear correlation was observed for the molar ratio, and the *K/S* value initially increased with the glycerol/citric acid ratio, reaching an optimum value at a molar ratio of 2:1. A further increase in the molar ratio, however, led to a decline in color strength. This can be attributed to the increased viscosity of the NaDES system at higher glycerol proportions, which hinders the diffusion and penetration of the NaDES into the PLA fibers, thereby reducing the treatment efficacy. Regarding the bath ratio, it exhibited minimal influence on the overall dyeing effect, and a ratio of 1:40 was sufficient for achieving satisfactory treatment efficacy.

Figure 4c indicates that the optimal dyeing performance was achieved when PLA fabric was treated with the NaDES at 80 °C. At this temperature, the treatment produced a roughened fiber surface, providing favorable sites for the adsorption of microbial prodigiosin, which enhanced dye uptake and yielded the maximum *K/S* value. In contrast, the NaDES treatment at 90 °C induced excessive surface etching and enhanced hydrophilicity, as confirmed by XPS analysis that showed a substantial increase in the surface oxygen content. These physicochemical changes reduced the adsorption capacity of PLA fibers for hydrophobic prodigiosin. Furthermore, the significant reduction in tensile strength observed for NaDES-treated PLA fabrics at 90 °C reflects a corresponding deterioration in fiber structural integrity, as depicted in Figure 4f. These findings demonstrate the necessity of balancing functional enhancement with the maintenance of material integrity during polymer modification [41]. Consequently, treatment at 80 °C achieves an optimal balance between moderate fiber modification and dye uptake, while the more aggressive treatment at 90 °C proves less effective.

With regard to the treatment time, Figure 4d indicates that the maximum *K/S* value was achieved at a treatment time of 90 min. Overall, the NaDES treatment significantly enhanced the dyeability of PLA fabric across all dyeing temperatures investigated. Remarkably, as shown in Figure 4e, the *K/S* value of the NaDES-treated PLA fabrics dyed at a low dyeing temperature of 70 °C surpassed that of the untreated fabric dyed at 90 °C, demonstrating the substantial potential of the NaDES treatment for energy-saving low-temperature dyeing.

**Figure 5:**
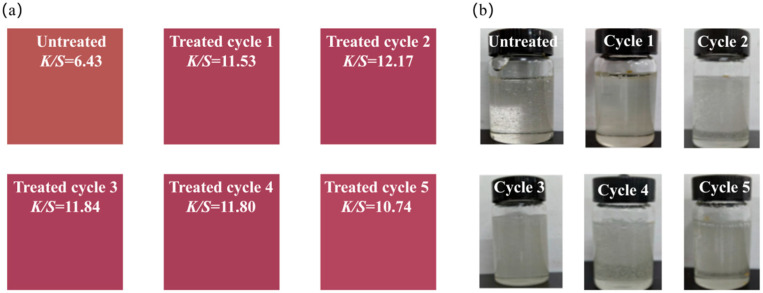
The images of dyed PLA fabrics (**a**) and NaDES state in different processing cycles (**b**).

From the perspectives of sustainable development and the practical economic viability of the NaDES system, evaluating its recyclability is essential for reducing chemical consumption and wastewater discharge. Figure 5 illustrate the changes in the dyeing performance of treated PLA fabrics and the status of the NaDES system after five recycling cycles. Overall, no significant decline in dyeing performance was observed for the treated PLA fabrics after five cycles, and the NaDES system remained homogeneous, colorless and transparent. This indicates that the developed NaDES system exhibits good stability and retention of treatment effectiveness during repeated use. However, during the recycling process, the NaDES system is subject to certain losses and residues due to its high viscosity, leading to a reduction in the concentration of effective components. Future research should therefore focus on the viscosity regulation of the NaDES system. By appropriately adding water or other functional additives to improve its fluidity, the recyclability of the system and the overall sustainability of the process can be further enhanced.

### 3.4. Mechanism Analysis

**Figure 6:**
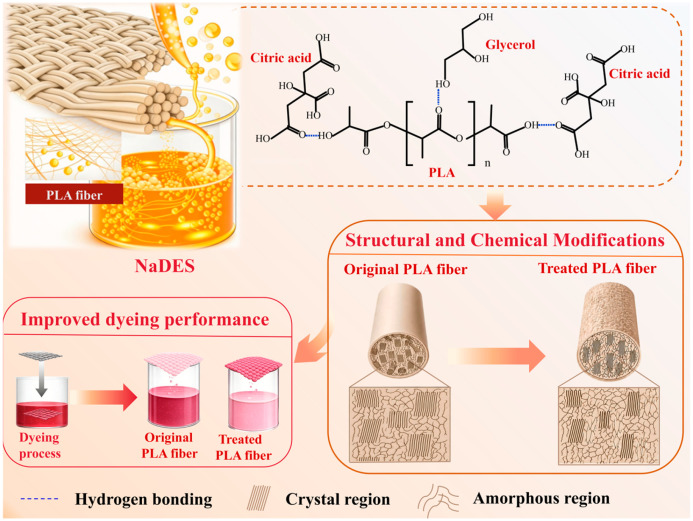
The mechanism of NaDES treatment on PLA fabrics.

Figure 6 elucidates the mechanism of NaDES treatment on PLA fibers, which improves dyeing performance through synergistic modifications at both the fiber surface and internal structure. As evidenced by the SEM images in Figure 3a–c, NaDES treatment induces discernible roughening of the PLA fiber surface, thereby providing additional sites for the adsorption of dye molecules. Regarding the internal structure, it has been reported that the NaDES can interfere with the hydrogen bonding network among PLA molecular chains, thereby influencing the crystal structure of the polymer [31,34]. Consistent with this, the FTIR data in Figure 3e and the XRD data in Figure 3f reveal that the penetration of the NaDES into the fiber interior disrupts the ordered arrangement of polymer chains through competitive hydrogen bonding. This interaction leads to a reduction in crystallinity and loosening of the internal fiber structure, thereby facilitating dye diffusion and enhancing dye uptake.

### 3.5. Antibacterial Properties

Given that prodigiosin exhibits remarkable antibacterial properties [42], the bacteriostatic rate of PLA fabrics dyed with microbial prodigiosin were evaluated against *S. aureus* and *E. coli*.

**Figure 7:**
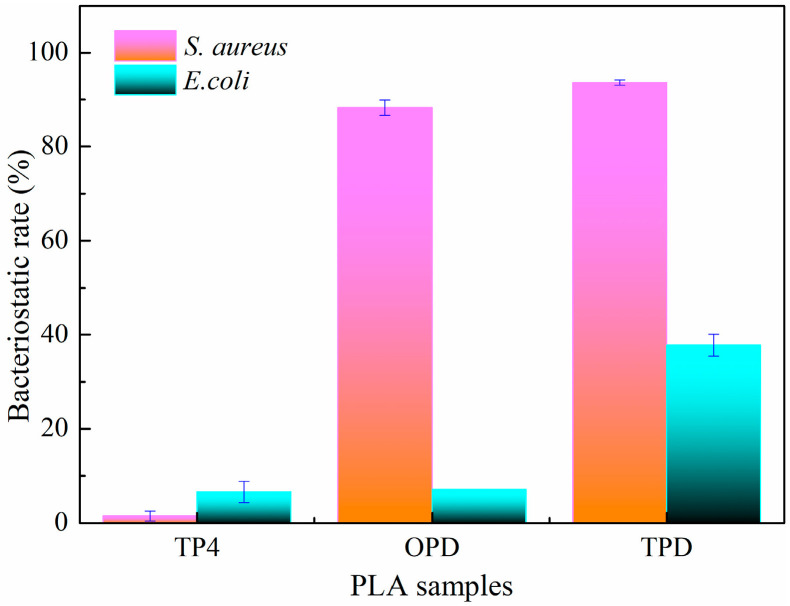
Bacteriostatic rate of PLA fabrics against *S. aureus* and *E. coli*. OPD is the original PLA fabric dyed with prodigiosin, while TPD is the NaDES-treated fabric dyed with prodigiosin.

As depicted in Figure 7, the PLA fabrics dyed with prodigiosin exhibited a favorable bacteriostatic rate against *S. aureus*, while a markedly weaker effect was observed against *E. coli*. The difference in antibacterial efficacy is primarily attributed to the distinct cell wall structure of Gram-positive and Gram-negative bacteria. The cell wall of *S. aureus*, a representative Gram-positive bacterium, consists of a thick, porous peptidoglycan layer that allows the relatively small prodigiosin molecules to penetrate readily and reach their intracellular targets. In contrast, the cell wall of *E. coli*, a typical Gram-negative bacterium, possesses a complex structure with an outer membrane external to the peptidoglycan layer. This outer membrane serves as an effective permeability barrier that restricts the penetration of hydrophobic molecules like prodigiosin, thereby reducing its antibacterial efficacy. Regarding the PLA samples dyed with prodigiosin, the bacteriostatic rate against *S. aureus* was 88.26% for OPD and 93.56% for TPD. The superior performance of the TPD sample can be attributed to the NaDES treatment, which enhanced the uptake of prodigiosin on the PLA fabric, thereby further improving its antimicrobial properties.

## 4. Conclusions

In summary, this study demonstrates that treatment with an NaDES composed of glycerol and citric acid could effectively improve the low-temperature dyeability of PLA fabrics. The modification mechanism involves both surface roughening and internal loosening of the PLA fibers. Surface roughening results from hydrogen bonding between the NaDES and PLA, together with the acid-catalyzed hydrolysis of ester bonds by citric acid. Internal loosening arises from NaDES penetration into the fibers, where hydrogen bonding leads to a reduction in crystallinity. These synergistic effects enhance the low-temperature dyeability of PLA fabrics. As a result, the accessibility and diffusion of microbial prodigiosin are improved, enabling the treated PLA fabric to achieve a high color depth at 70 °C, comparable to that of untreated PLA fabrics dyed at 90 °C. This can effectively reduce the energy consumption input and carbon emissions during the dyeing process, meeting the requirements of the green and sustainable development of the textile industry. Simultaneously, the low-temperature dyeing process preserved the antibacterial effect of prodigiosin, and the bacteriostatic rate of the dyed PLA fabric against *S. aureus* reached 93.56%. This approach provides a promising eco-friendly alternative for functional textile dyeing. Future research could focus on the rational design of NaDES systems, with emphasis on overcoming the limitations caused by high viscosity, so as to further improve the treatment efficiency during the modification and dyeing of PLA fabrics. Meanwhile, as a green and biodegradable solvent, the NaDES aligns with the sustainable development goals of the textile industry. Its recovery and recycling can further reduce solvent consumption and production costs, thereby facilitating its industrial application in line with circular economy principles.

## Data Availability

The data presented in this study are available on request from the corresponding author.
